# Identification of a mammalian silicon transporter

**DOI:** 10.1152/ajpcell.00219.2015

**Published:** 2017-02-08

**Authors:** Sarah Ratcliffe, Ravin Jugdaohsingh, Julien Vivancos, Alan Marron, Rupesh Deshmukh, Jian Feng Ma, Namiki Mitani-Ueno, Jack Robertson, John Wills, Mark V. Boekschoten, Michael Müller, Robert C. Mawhinney, Stephen D. Kinrade, Paul Isenring, Richard R. Bélanger, Jonathan J. Powell

**Affiliations:** ^1^Medical Research Council Elsie Widdowson Laboratory, Cambridge, United Kingdom;; ^2^Department of Veterinary Medicine, University of Cambridge, Cambridge, United Kingdom;; ^3^Département de Phytologie-Faculté des Sciences de l'Agriculture et de l'Alimentation, Centre de Recherche en Horticulture, Université Laval, Quebec City, Quebec, Canada;; ^4^Department of Zoology, University of Cambridge, CambridgeUnited Kingdom;; ^5^Institute of Plant Science and Resources, Okayama University, Kurashiki, Japan;; ^6^Mechanistic Studies Division, Environmental Health Sciences & Research Bureau, Health Canada, Ottawa, Ontario, Canada;; ^7^Nutrition, Metabolism and Genomics Group, Division of Human Nutrition, Wageningen University, Wageningen, The Netherlands;; ^8^Department of Chemistry, Lakehead University, Thunder Bay, Canada; and; ^9^Nephrology Group L’Hôtel-Dieu de Québec Institution, Department of Medicine, Faculty of Medicine, Université Laval, Quebec City, Quebec, Canada

**Keywords:** silicon, transport, Slc34a2, *Xenopus laevis* oocytes, rat kidneys

## Abstract

Silicon (Si) has long been known to play a major physiological and structural role in certain organisms, including diatoms, sponges, and many higher plants, leading to the recent identification of multiple proteins responsible for Si transport in a range of algal and plant species. In mammals, despite several convincing studies suggesting that silicon is an important factor in bone development and connective tissue health, there is a critical lack of understanding about the biochemical pathways that enable Si homeostasis. Here we report the identification of a mammalian efflux Si transporter, namely Slc34a2 (also termed NaPiIIb), a known sodium-phosphate cotransporter, which was upregulated in rat kidney following chronic dietary Si deprivation. Normal rat renal epithelium demonstrated punctate expression of Slc34a2, and when the protein was heterologously expressed in *Xenopus laevis* oocytes, Si efflux activity (i.e., movement of Si out of cells) was induced and was quantitatively similar to that induced by the known plant Si transporter *Os*Lsi2 in the same expression system. Interestingly, Si efflux appeared saturable over time, but it did not vary as a function of extracellular $HPO_{4}^{2-}$ or Na^+^ concentration, suggesting that Slc34a2 harbors a functionally independent transport site for Si operating in the reverse direction to the site for phosphate. Indeed, in rats with dietary Si depletion-induced upregulation of transporter expression, there was increased urinary phosphate excretion. This is the first evidence of an active Si transport protein in mammals and points towards an important role for Si in vertebrates and explains interactions between dietary phosphate and silicon.

silicon (Si) is the second most abundant element in the Earth’s crust and is ubiquitous in the diet, but the role it plays in mammalian physiology remains unclear. There is substantial evidence for its importance in the normal health and development of bone and connective tissues of vertebrates (6, 25, 43, 45), but a specific physiological and/or metabolic function has not been identified. In particular, proteins responsible for Si transport in mammals remain elusive. Silicon is essential for many algae (e.g., diatoms) to produce their exoskeleton and to complete their cell cycle (5, 21). It is also important in many species of plants, with both structural and physiological roles identified (12, 13, 27).

The first Si transporter to be identified (*Cf*SIT1) was in the diatom species *Cylindrotheca fusiformis* (22), and SITs are now known from a wide range of diatoms (51), choanoflagellates (32), and haptophytes (11). In plants, Si transport occurs through a collaboration of two individual transporter types, one of which is responsible for influx (movement of Si into cell) and the other for efflux (movement of Si out of cell). Influx occurs through an aquaporin channel (e.g., Lsi1, Lsi6) whereas efflux occurs through an energy-dependent active transport process driven by a proton gradient (e.g., Lsi2) (29, 30). Despite the characterization of multiple Si transporters in algae and plants as described, no Si-transporting homologs have been reported in mammals yet (29, 30, 32).

Previously, we reported that Sprague-Dawley rats on a Si-depleted diet massively reduced their urinary Si output to maintain serum and tissue Si levels (24). This was at the expense of phosphorus, which was decreased in serum and bone (24). These findings suggested that the kidney may be actively involved in Si conservation under chronic Si deprivation and that, somehow, phosphate is lost in the process. Here we report on the mammalian phosphate transport protein Slc34a2, which was upregulated in the kidney of the rats deprived of dietary Si. This protein was found to induce Si efflux activity when expressed in *Xenopus* oocytes and to exhibit structural similarity with Lsi2 in many plants. Identification that Slc34a2 can transport Si provides new evidence for a biological role for this element in mammals and establishes another distinct gene family of Si transporters.

## MATERIALS AND METHODS

### Silicon Depletion Study

Kidneys were obtained from the study of Jugdaohsingh et al. (24) following 6 mo of dietary Si intervention. Three-week-old female Sprague-Dawley rats were maintained for 26 wk on a formulated low-Si feed (~3 µg Si/g feed), with either low-Si drinking water (~15 ng Si/g water; Si deplete group, *n* = 20) or with orthosilicic acid (H_4_SiO_4_) supplemented in the drinking water (53 µg Si/g water; Si replete group, *n* = 10). A reference group of rats received a normal laboratory maintenance chow diet (B&K Rat and Mouse Standard Diet; B&K Universal) which is naturally high in Si (322 µg Si/g feed) and tap water (5 µg Si/g water); see reference 24 for diet compositions. This third group of rats is referred to as Si-high reference group. Total Si intakes were 0.17 mg Si kg body wt^−1^·day^−1^ in the Si deplete group, 4.1 mg Si·kg^−1^·day^−1^ in the Si replete group, and 18.5 mg Si·kg^−1^·day^−1^ in the Si-high reference group. After 26 wk, rats were euthanized by asphyxiation with carbon dioxide gas as previously described (24). Rats were killed and processed one at a time, with one rat from each group on the same day. Tissues were then harvested, as previously described (24), and stored at −20°C immediately following harvesting and then at −80°C for long-term storage. All groups of rats and their tissues were treated in precisely the same fashion. Spot urine samples were collected from fasted rats (24). Urinary Si and P analysis was by inductively coupled plasma optical emission spectrometry (ICP-OES), as described below, and data were corrected for urinary creatinine (24). As previously described (24), all animal procedures were carried out in accordance with the UK Home Office Animal Scientific Procedures Act 1986 (Scientific Procedures on Living Animals). Use of laboratory animals was approved by King’s College London (UK) Animal Ethics Committee and the UK Home Office. For this study, the left kidneys from *n* = 10 Si deplete, *n* = 8 Si replete, and *n* = 5 Si-high reference rats were ground in liquid nitrogen and total RNA was extracted with the Qiagen RNeasy Maxi kit for microarray and quantitative PCR analysis. This part of the study was carried out in 2008.

### Gene Array Analysis

Five micrograms total RNA per sample were hybridized to Affymetrix GeneChip Rat Genome 230 2.0 arrays (*n* = 4 Si deplete and *n* = 4 Si replete kidneys). Gene chip robust multiarray analysis (gcRMA) was used to normalize the data including a summarization step based on m-estimator values for the probe sets (58). Modified T-statistics were used to calculate significance of differential gene expression (10, 44) between the Si replete vs. Si deplete groups. Genes were selected as “differentially expressed” when false discovery rate *q* was < 0.1 (49). [The microarray data set has been submitted to the NCBI Gene Expression Omnibus: accession no: GSE58404.]

### Expression Studies

Quantitative real-time PCR was used to investigate the expression of relevant transcripts, including that of an internal control (18S), in the full cohort of rat kidney RNAs (*n* = 10 Si deplete, *n* = 8 Si replete, and *n* = 5 Si-high reference group). Transcripts were amplified with the TaqMan Universal protocol for real-time RT-PCR. The primers were TaqMan Gene Expression Assays consisting of a FAM reporter and TaqMan MGB probes. Differences in gene expression between groups were statistically analyzed by unpaired *t*-test.

### Immunohistochemistry

Kidneys from a normal laboratory maintenance chow fed rat were excised immediately after necropsy and then fixed in 4% PBS-buffered paraformaldehyde. The samples were then cryo-protected via sucrose gradient and snap-frozen in iso-pentane cooled on dry ice. The frozen samples were then embedded in Optimal Cutting Temperature compound (VWR). Tissue sections were subsequently cryo-sectioned at 12 μm thickness, collected on SuperFrost slides (Thermo Scientific), and allowed to air dry overnight at room temperature. Sections were blocked with normal serum in PBS. Samples were then incubated with primary antibody against Slc34a2 (Genetex) or an appropriately matched isotype control. Primary antibody was then detected by incubation with goat anti-rabbit IgG (H+L) Alexa Fluor 488 conjugate (Invitrogen) before counterstaining the nuclei with Hoechst 33342 (Invitrogen) and the cytoskeleton (f-actin) with phalloidin CF633 (Biotium). Imaging was carried out on a Leica SP2 confocal microscope using a 1.2 numerical aperture ×63 water immersion lens. Images were collected using the Leica Application Suite software. Alongside Slc34a2 antibody-stained sections, images of the isotype controls were also collected under identical settings and in “matched” parallel tissue sections. A threshold removing any minor nonspecific signal in the isotype controls was then defined, with this threshold subsequently applied identically across all collected images to robustly identify Slc34a2. Staining for Slc34a2 was distinctly punctate so, as well as presentation in as-collected intensity format, images are also presented in binary format (i.e., all Slc34a2 signal that is brighter than isotype threshold shown at maximum intensity). This “view” was included to facilitate visualization of Slc34a2 locality within the limits of the printed image size.

### Urinary P and Si Analyses

Fasting spot urine samples collected from 6-h fasted rats (*n* = 8 Si deplete, *n* = 5 Si replete, and *n* = 6 Si-high reference rats) were digested (in 1:1 mixture of 69% nitric acid and 40% hydrogen peroxide), diluted (1:100), and analyzed for total phosphorus by inductively coupled plasma optical emission spectrometry (ICP-OES; Jobin Yvon 2000-2) at 214.914 nm with sample-based standards. Urinary Si was analyzed by ICP-OES as previously described (24).

### Inter-Organism Homology of Si Transporters

Homology search was performed with BLASTp (3) against plant and diatom sequences in the EMBL/GenBank nonredundant protein database using the default settings (http://www.ncbi.nlm.nih.gov/). BLASTp and tBLASTn were also used to identify homologs in a range of fully sequenced vertebrate genomes from the EMBL/GenBank and Ensembl databases, and also to identify homologs in selected phylogenetically relevant groups where complete genomes were not available (see Supplemental Table S1; Supplemental Material for this article is available at the Journal website). An alignment of homologs was generated using MUSCLE (http://www.ebi.ac.uk/Tools/msa/muscle/) under the default settings, producing a final alignment of 38 sequences from 17 species. ProtTest (1) found that the JTT+G+I model provided the best fit to the data under the Akaike Information Criterion. Maximum likelihood analysis was carried out using PhyML (19). Starting trees were generated by BioNJ, with tree searching using the NNI heuristic methods, and topology and branch lengths were optimized in ML calculations. One hundred bootstrap data sets were analyzed using the same model and method as for the PhyML analysis, with bootstrap proportions added as numbers to the nodes of the PhyML tree. The alignment was also used for Bayesian MCMC analysis using Phylobayes 3.3 software (26), under the CAT +G+I model until convergence (maximum discrepancy <0.3, effective size >100), for 15 parallel chains with sampling every 100 cycles and a burn-in equal to one-fifth the total size of the chain. Posterior probabilities were used to express the support for the nodes in the Bayesian phylogeny. The trees generated were viewed using FigTree (version 1.3.1, Andrew Rambaut, Institute of Evolutional Biology, University of Edinburgh 2006–2009).

### Calculated Oxoacid Volumes

The structure for each oxoacid/oxoanion was optimized using the PBE0 functional (2, 38, 39) and 6–31++g(d,p) atomic orbital basis set (4, 9, 15, 16, 20, 41, 42). The electron density corresponding to these optimized structures was used to estimate the molecular volume that describes the solvent accessible surface, defined as the volume bounded by a density contour of 0.001 electrons/Bohr^3^. An increased density of points was used to ensure a more accurate integration so that the computed molecular volumes are quantitative (37, 56). Since these species are in an aqueous environment, structures were optimized within a solvent field using the integral equation formalism variant of the polarizable continuum model (7, 52, 57) to account implicitly for the effects of an aqueous environment on the solvent accessible surface. The Gaussian09 suite of programs (17) was used in these determinations.

### Transport Activity in Xenopus laevis Oocytes

#### Cloning the gene of interest and oocyte preparation.

A cDNA sequence verified *Rattus norvegicius* IMAGE clone pExpress-1/Slc34a2 (Unigene ID: Rn.16933, Entrez Gene: 84395 in DH10BTonA) was purchased from Source BioScience LifeSciences (Cambridge, UK).

For synthesis of capped RNA, the open reading frame (ORF) was amplified by PCR with the following primers: 5′-GAGGATCCATGGCTCCTTGGCCCGAGTTG-3′ and 5′-GAGGATCCTAGAACACTGTAGTGTTGGACA-3′. The fragment containing the ORF was inserted into the *Bgl*II site of a *Xenopus* oocyte expression vector pXßG-ev1 (a pSTP64 T-derived pBluescript type vector into which *Xenopus* β-globin 5′- and 3′-UTR had been inserted) (40). Capped RNA was then synthesized from linearized pXβG-ev1 plasmids by in vitro transcription with mMESSAGE mMACHINE High Yield Capped RNA Transcription Kit (Ambion) according to the manufacturer’s instructions.

Oocytes were isolated from *Xenopus laevis* frogs purchased from NASCO (Nasco-Fort Atkinson, WI) and from Watanabe Zosyoku (Hyogo Pref, Japan). Procedures for defolliculation, culture condition, and selection were the same as described previously (35). A volume of 50 nl of the in vitro cRNA transcripts (1 ng/nl) was injected into stage V oocytes using a Nanoject II automatic injector (Drummond Scientific). Water-injected oocytes were used as a negative control, *Os*Lsi1-injected oocytes were used as positive controls while testing for influx activity, and *Os*Lsi2-injected oocytes were used as positive controls while testing for efflux activity. Ethical approval was obtained (permit no. 21031043) from the Animal Care Committee of Laval University (Quebec City, QC, Canada).

#### Influx transport activity.

After incubation in a Modified Barth’s Saline (MBS) solution (88 mM NaCl, 1 mM KCl, 2.4 mM NaHCO_3_, 15 mM Tris·HCl at pH 7.6, 0.3 mM Ca(NO_3_)_2_, 0.41 mM CaCl_2_, 0.82 mM MgSO_4_, 10 μg/ml sodium penicillin, and 10 μg/ml streptomycin sulfate) at 18°C overnight, the cRNA-injected oocytes were exposed to the MBS solution supplemented with 1 mM H_4_GeO_4_, 0.1 mM $HAsO_{4}^{2-}$ or 1 mM $HPO_{4}^{2-}$ at pH 7.6. Following 30 min incubation at 18°C, the oocytes were washed five times with MBS alone and digested with concentrated (61%) HNO_3_. The Ge, As, and P concentrations in the digested solutions were determined by ICP-MS (7700X; Agilent Technologies) with appropriate standards, QCs and sample blanks.

To investigate the Si influx and its dependence on extracellular [Na^+^] or [$HPO_{4}^{2-}$], oocytes were incubated for three days at 18°C in MBS5 (84 mM Na^+^ and 2 mM $HPO_{4}^{2-}$) supplemented with 100 µM each of penicillin and streptomycin. Then a set of 10 oocytes for each condition was exposed to MBS2 (1.7 mM H_4_SiO_4_, 10 mM Na^+^, and 0.5 mM HP$HPO_{4}^{2-}$) or MBS3 (1.7 mM H_4_SiO_4_, 84 mM Na^+^, and 2 mM $HPO_{4}^{2-}$) solution for 2 h. After exposure, oocytes were rinsed in a solution containing 0.32 M sucrose and 5 mM HEPES (pH 7.4) and then digested in 25 µl concentrated nitric acid, dried at 82°C for 2 h, reconstituted in plasma grade water (100 µl) and 10 µl analyzed by atomic absorption spectroscopy (see below).

#### Efflux transport activity.

To investigate the efflux transport activity for H_4_GeO_4_ by *Rn*Slc34a2, 50 nl 1 mM H_4_GeO_4_ in MBS were directly injected into *Rn*Slc34a2-transfected oocytes. The oocytes were then washed five times with MBS and transferred to 200 µl of fresh MBS at 18°C. H_4_GeO_4_ was allowed to efflux into the incubation medium. After 30 min and 2 h, the incubation medium was carefully sampled, and at the end of the experiment, the oocytes were digested with concentrated HNO_3_ and the samples were analyzed for Ge by ICP-MS (7700X) with appropriate standards, QCs, and sample blanks.

To investigate the efflux transport activity for Si by *Rn*Slc34a2, oocytes were injected with 25 nl of 500 ng/nl cRNA of *RnSlc34a2* or *OsLsi2* or an equal volume of H_2_O as a negative control. Pools of 10 oocytes were then loaded with Si by incubation for three days at 4°C in MBS1 or MBS2, both containing 2 mM Si but different concentrations of Na^+^ and $HPO_{4}^{2-}$ (Supplemental Table S2). These oocytes were then exposed to fresh MBS without added Si but with different concentrations of Na^+^ and $HPO_{4}^{2-}$ (MBS, MBS3, MBS4, or MBS5 solution; Supplemental Table S2) for 0, 1, or 2 h. After exposure, oocytes were rinsed in a solution containing 0.32 M sucrose and 5 mM HEPES (pH 7.4), digested with concentrated HNO_3_ (25 µl for each pool of 10 oocytes) and dried at 82°C for 2 h. Plasma-grade water (100 µl) was then added, and samples were incubated for 1 h at room temperature. Samples were vortexed and then centrifuged for 5 min at 13,000 *g*. Ten microliters of samples were then analyzed by Zeeman atomic absorption spectrometer (Varian AA240Z; http://www.varian.com) equipped with a GTA120 Zeeman graphite tube atomizer, to determine the intracellular Si concentration. Silicon levels in the samples and sample blanks were quantified using appropriate standards prepared using 1,000 ppm ammonium hexafluorosilicate solution (Fisher Scientific, http://www.fishersci.com). Data were analyzed with SpectrA software (Varian).

### Statistical Analysis

Results are reported as means ± SD unless otherwise stated. Linear relationships between dietary Si exposure and the relative renal expression of Slc34 genes were assessed at a significance of *P* ≤ 0.05. Thereafter, individual differences were assessed by Independent (unpaired) Samples 2-Tailed T-test. Where there were multiple group comparisons a Bonferroni correction was applied to the *P* value (i.e., *P*/*n*), and significance was taken as *P* ≤ 0.05/*n*. All statistical analysis was conducted in GraphPad Prism (version 6.0b) or IBM SPSS software (version 21; IBM).

## RESULTS AND DISCUSSION

### Identifying RnSlc34a2 As a Candidate for Si Transport

**Fig. 1.**
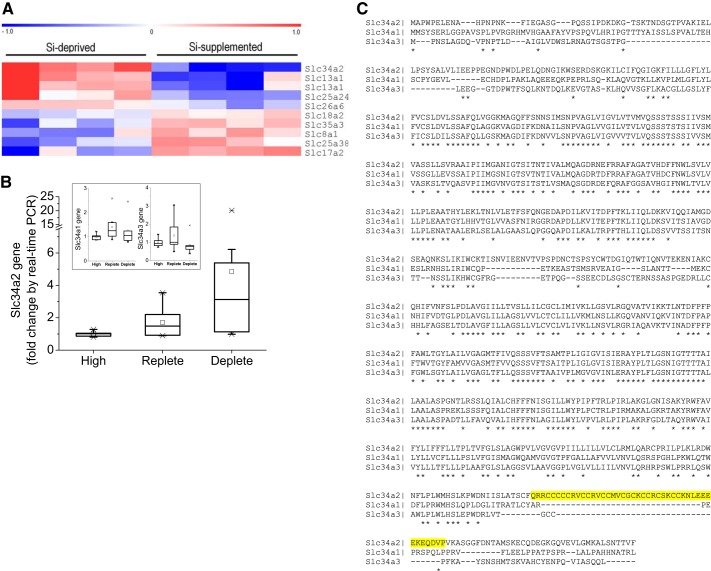
Identifying *Rn*Slc34a2 as a candidate for Si transport. *A*: relative expression of solute-like carriers in the kidney of *Rattus norvegicus* from Si deplete (*n* = 4) compared with Si replete (*n* = 4) animals. Data were analyzed by gene array. Red indicates upregulation and blue indicates downregulation of expression; Si replete vs. Si deplete group. Multiple probe sets per gene can be present as was the case for Slc13a1. *B*: quantitative PCR analysis of Slc34a2 and of family members (*inset*) in the kidneys of Si-high reference, Si replete and Si deplete rats. Overall, the relative expression of *Rn*Slc34a2 was inversely related to dietary Si exposure (*P* < 0.05), but there was no relationship with Slc34a1 or Slc34a3 (*P* = 0.5 and 0.4, respectively). Gene expression values are relative to the Si-high reference group. *C*: sequence alignment of the *Rattus norvegicus* Slc34 gene family. Slc34a2 is characterized by a ~30-residue stretch (highlighted in yellow) that is not present in Scl34a1 and Slc34a3. Asterisks (*) below sequence indicate identical amino acids, colons (:) indicate functionally similar amino acids, and dashes (–) indicate gaps in the alignment.

Data from our study of Si deficiency in rats strongly suggested active urinary conservation of Si during dietary Si depletion (24). The present investigation utilized kidneys harvested during this study to investigate Si regulatory genes. Gene arrays (Affymetrix GeneChip Rat Genome 230 2.0 arrays containing over 16,000 Entrez IDs and 11 probes per gene) were performed on RNA extracted from the kidney tissues of Si deplete and Si replete rats (*n* = 4 for each group) and the data were interrogated for differential regulation of potential transporters (Fig. 1*A*). The gene array findings^1^ were confirmed by real-time RT-PCR analysis, and this technique was also subsequently used to investigate a larger cohort of samples from the Si deplete (*n* = 10), Si replete (*n* = 8), and a reference group (*n* = 5) that were rats kept on a normal laboratory chow diet that is naturally high in Si (referred to as Si-high reference group). Slc34a2 (type II sodium-phosphate cotransporter), commonly referred to as NaPi-IIb, was expressed especially highly in the kidneys of rats on the Si deplete diet (2.8- and 4.8-fold higher than for kidneys from rats on the Si replete and Si-high reference diets, respectively; Fig. 1*B*). mRNA expression of other Slc34 family members, namely Slc34a1 and Slc34a3, were unchanged with dietary Si intervention (*inset*, Fig. 1*B*).

**Fig. 2.**
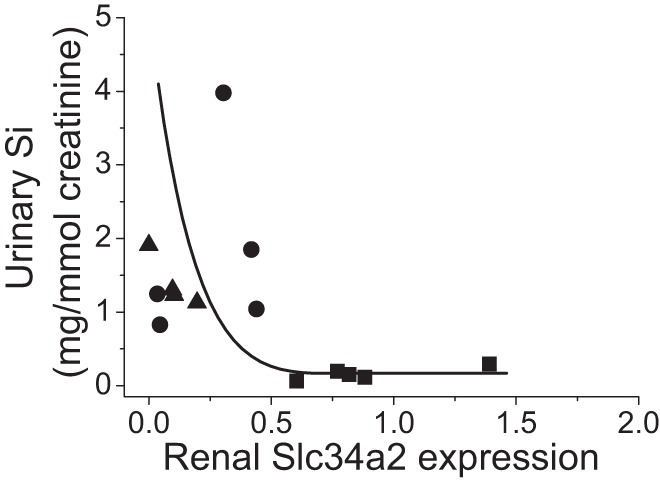
Correlation between renal *Rn*Slc34a2 expression (by quantitative RT-PCR analysis) and fasting urinary Si excretion. Urinary Si excretion in the rats (■, Si deplete; ●, Si replete) and laboratory chow reference group (▲, Si-high reference) showed an inverse relationship with Slc34a2 expression in the kidneys; *r* = 0.47.

Correlation between Slc34a2 expression and urinary Si concentration showed an inverse exponential relationship between fasting urinary Si level and the relative expression of Slc34a2 (Fig. 2), implying that Slc34a2 is involved in the reabsorption of H_4_SiO_4_ from the pre-urine under dietary Si deprivation. No such relationship was observed for Slc34a1 and Slc34a3, or other candidate transporters identified in the gene arrays (Fig. 1*A*).

**Fig. 3.**
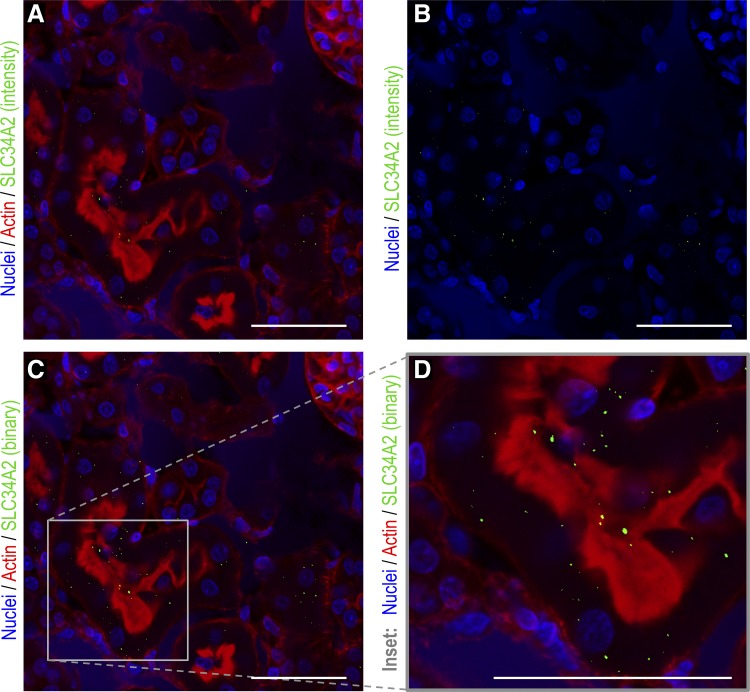
Immunohistochemistry analysis of Slc34a2 in freshly harvested rat kidney cortex. Sections of freshly harvested kidneys from a healthy wild-type rat were analyzed by immunohistochemistry with anti-Slc34a2 (*green*) antibody (this figure) or the appropriate isotype control (data not shown). Cell nuclei were counterstained (*blue*) with Hoescht 33342 and cell cytoskeleton (f-actin, *red*) with phalloidin CF633. Antibody-stained sections and isotype controls were collected under identical settings, as stated in materials and methods. A threshold removing all Slc34a2 attributable signal was defined on the isotype controls and uniformly applied to all images (i.e., antibody-stained images). Staining for Slc34a2 within the tubular epithelial cells was distinctly punctate so, as well as the signal above the isotype control being presented in an as-collected “intensity” format (i.e., the more secondary antibody that is bound, the brighter the signal) (*A* and *B*), it is also displayed as a binary format (i.e., all signal that is brighter than isotype threshold is given the maximum intensity value) as this aids visualization (*C* and *D*). All images are of the kidney cortex and scale bars are 50 µm. *B*: as-collected “intensity” format without actin staining. *D*: a high-power image (×63 magnification) of the area within the quadrant in image (*C*).

Only a few reports have demonstrated the renal expression of Slc34a2. The original paper characterizing the transporter demonstrated its presence in murine kidney at the mRNA level (23). Suyama et al. (50) confirmed this more recently by in situ hybridization as well as protein expression and localization by antibody staining. The kidney samples from our study were not adequately collected for immunohistochemical analysis, but were for RNA analysis. Thus we confirmed with appropriately collected kidneys from a control rat that, as previously published (50), Slc34a2 protein is expressed by the tubular epithelial cells of the kidney cortex (Fig. 3). Here, as previously reported (50), Slc34a2 showed distinct punctate staining: some of which was basolateral within the cell and some of which was apical/cytosolic (Fig. 3). Whether silicate deficiency dictates only the level of expression of Slc34a2 (Fig. 1) or, also its precise location in the cell, as excess dietary phosphate does (50), should be investigated in future work.

**Fig. 4.**
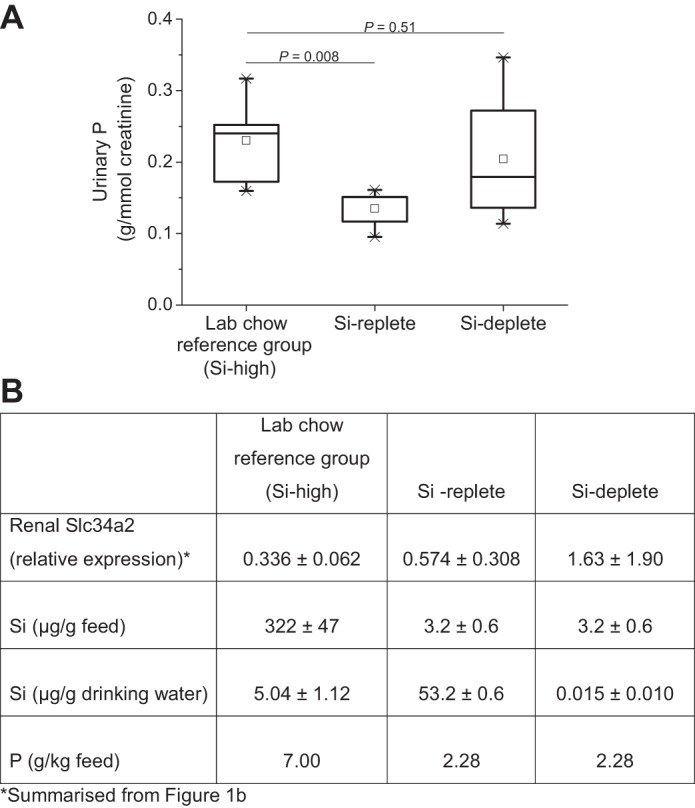
Fasting urinary phosphorus excretion. Urinary P excretion was measured in the laboratory chow reference group (Si-high reference; *n* = 6), Si replete (*n* = 5), and Si deplete (*n* = 8) rats by ICP-OES and corrected for creatinine concentration (*A*). The higher P excretion in the laboratory chow reference group is due to the higher P content of the diet (see *B*). However, the difference in urinary P excretion between the Si replete and Si deplete rats cannot be explained by a difference in dietary P content, but rather due to the upregulation of Slc34a2 in the latter group mediated by Si deficiency in the diet and drinking water (*B*).

Finally, to translate these observations (i.e., that Slc34a2 has some basolateral expression in kidney cells and is upregulated in Si deplete diets) we measured urinary P excretion in the three groups. The Si-high reference group diet was higher in P than the Si replete group diet, being 7.0 vs. 2.3 mg/g, respectively, and so, as expected, urinary P excretion was significantly reduced in the latter [by 89 mg/mmol creatinine for the medians; *P* = 0.008; *n* = 6 and 5, respectively (Fig. 4)]. However, the Si deplete group (with the same dietary P level as the Si replete group) showed no difference in urinary P levels compared with the reference group (Fig. 4), showing that in this group, phosphate was being (relatively) wasted as a consequence of Si being conserved.

### RnSlc34a2 Transport Activity

The ubiquitous nature of Si makes transport studies of soluble silicic acid (H_4_SiO_4_) challenging. It is well known that related oxoacids may ride the same transport systems (22, 29, 36, 54) owing to similarities in their structure and solvated molecular volume (Table 1). Germanic acid (H_4_GeO_4_), the closest structural analog of silicic acid, is therefore often employed as a proxy for Si transport, thereby avoiding all background and contamination issues with Si and facilitating analysis (22, 29). Recently, however, graphite furnace atomic absorption spectrometry (GFAAS) was shown to be effective for directly measuring Si influx/efflux in *Xenopus laevis* oocytes transfected with Si-transporting aquaporins from plants (8, 18). Hence, both methods of characterizing Si transport— indirect and direct—were used in the present investigation.

**Table 1.** Oxoacid and oxoanion volumes

Oxoacid/Oxoanion	Gas Phase V_M_, cm^3^/mol	Implicit Aqueous Solvent V_M_, cm^3^/mol
$HCO_{3}^{-}$	41.9	39.1
H_3_BO_3_	43.4	43.6
$H_{4}BO_{4}^{-}$	52.5	55.8
H_4_SiO_4_	63.2	56.5
H_4_GeO_4_	61.3	57.8
$HPO_{4}^{2-}$	62.0	59.2
$HAsO_{4}^{2-}$	65.0	63.1

PBE0/6–31++g(d,p) calculated solvent accessible surface molar volumes (V_M_) for various main group oxoacids and oxoanions at physiological pH, showing the similarities between H_4_SiO_4_, H_4_GeO_4_ (two oxoacids that are effluxed by Slc34a2), $HPO_{4}^{2-}$, and $HAsO_{4}^{2-}$ (oxoanions that are influxed by Slc34a2).

**Fig. 5.**
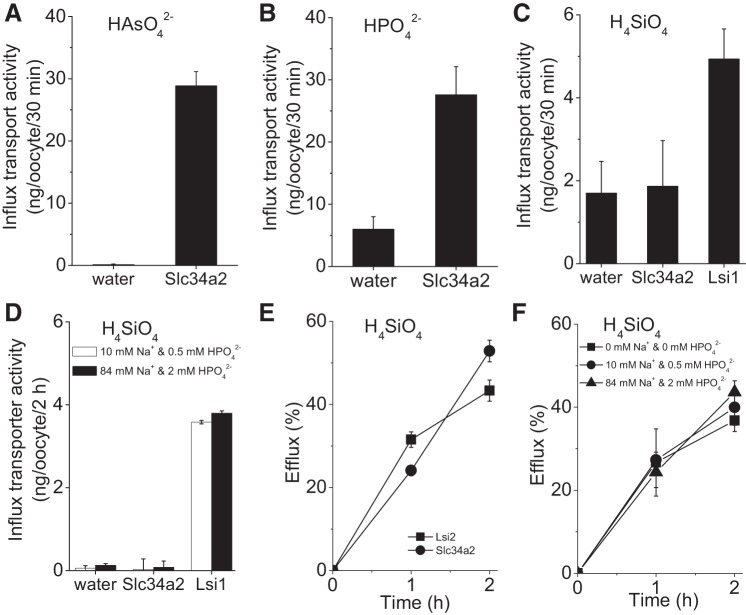
Transport activity in *Rn*Slc34a2-expressing oocytes. *A–C*: influx transport activity of *Rattus norvegicus* Slc34a2 for arsenate, $HAsO_{4}^{2-}$ (*P* = 0.0001) (*A*), phosphate, $HPO_{4}^{2-}$ (*P* = 0.0008) (*B*), and silicic acid, H_4_SiO_4_ (*P* = 0.66) (*C*) . Rice transporter Lsi1 was used as a positive control for H_4_SiO_4_ influx (*P* < 0.0001). *D*: the concentrations of sodium and phosphate in the medium did not influence H_4_SiO_4_ influx by *Rn*Slc34a2-expressing oocytes, nor that by *Os*Lsi1-expressing oocytes (*P* < 0.0001 in both instances). Water-injected oocytes were used as a negative control. *E*: in H_4_SiO_4_ efflux studies, rice transporter Lsi2 was used as a positive control. Data were corrected against water-injected control oocytes. *F*: changes in sodium and phosphate concentration did not affect H_4_SiO_4_ efflux by Slc34a2 expressing oocytes. Data are shown as means ± SE (*n* = 15).

**Fig. 6.**
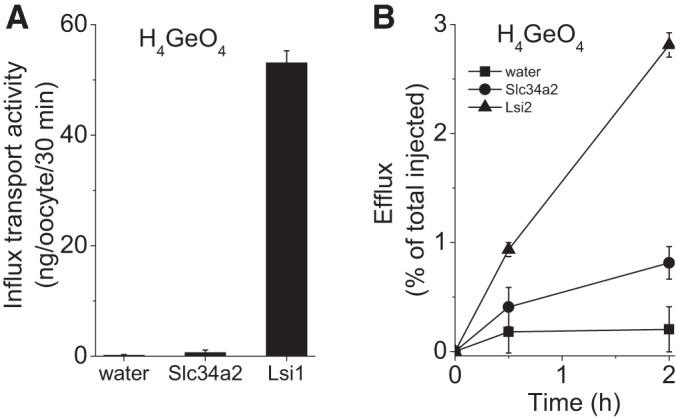
Germanium transport activity in *Rn*Slc34a2-expressing oocytes. Transport activity for H_4_GeO_4_ showing a lack of influx (*P* = 0.14) (*A*) but significant efflux (*B*) following a 2 h incubation (*P* = 0.004). Efflux was not significant at 30 min (*P* = 0.14) for Slc34a2-expressing oocytes. The rice Si transporters Lsi1 and Lsi2 were used as positive controls for influx and efflux activity, respectively (*P* < 0.0001 in both cases compared with negative control, water-injected oocytes).

The Slc34a2 coding sequence was inserted into a *Xenopus laevis* expression vector, and cRNA synthesized from this construct was injected into oocytes. Initial expression and plasma membrane localization were verified using an eGFP-tagged Slc34a2 construct. Slc34a2 is recognized as a sodium phosphate importer, especially in the brush border of small intestine membrane cells (14, 31, 36, 55), and arsenate also rides this transport system (36, 54). Therefore, both of these oxoacids were utilized as easily measured probes to confirm Slc34a2 influx activity (Fig. 5, *A* and *B*). The rice Si importer *Os*Lsi1 was used as a positive control and was found to promote both H_4_SiO_4_ (Fig. 5*C*) and H_4_GeO_4_ (Fig. 6*A*) influx. By contrast, no influx of either H_4_SiO_4_ or H_4_GeO_4_ by Slc34a2-expressing oocytes was observed (Figs. 5*C* and 6*A*). On the other hand, efflux of both H_4_SiO_4_ and H_4_GeO_4_ was detected for oocytes expressing Slc34a2 (Figs. 5*E* and 6*B*, respectively) as well as those expressing rice Si exporter *Os*Lsi2, which was employed as a positive efflux control. Of note, the magnitude of fractional H_4_SiO_4_ efflux after 2 h was quantitatively similar between Slc34a2 and *Os*Lsi2.

Given that inward phosphate ($HPO_{4}^{2-}$) transport by Slc34a2 is coupled to the inward transport of three sodium ions [i.e., it is electrogenic (14, 55)], we investigated how varying the concentrations of Na^+^ and $HPO_{4}^{2-}$ in the external medium might influence Si influx and efflux in Slc34a2-expressing oocytes. No significant effects were observed at the broad concentrations investigated (Figs. 5, *D* and *F*). These findings suggest that Si is not translocated across the membrane through the Na^+^ or $HPO_{4}^{2-}$ transport site, but through an independent transport site that is potentially involved in Si efflux primarily. In keeping with this possibility is the presence of multiple, often independent binding sites in a number of ABCD family members (47). Alternatively, Slc34a2 could cooperate with accessory proteins to promote Si efflux. In this regard, the Na^+^-K^+^-ATPase γ-subunit FXYD2 appears to play a role in Mg^2+^ transport, while the α- and β-subunits alone do not exhibit such transport capabilities (46).

### Homology Between Slc34a2 and Si Transporters

**Fig. 7.**
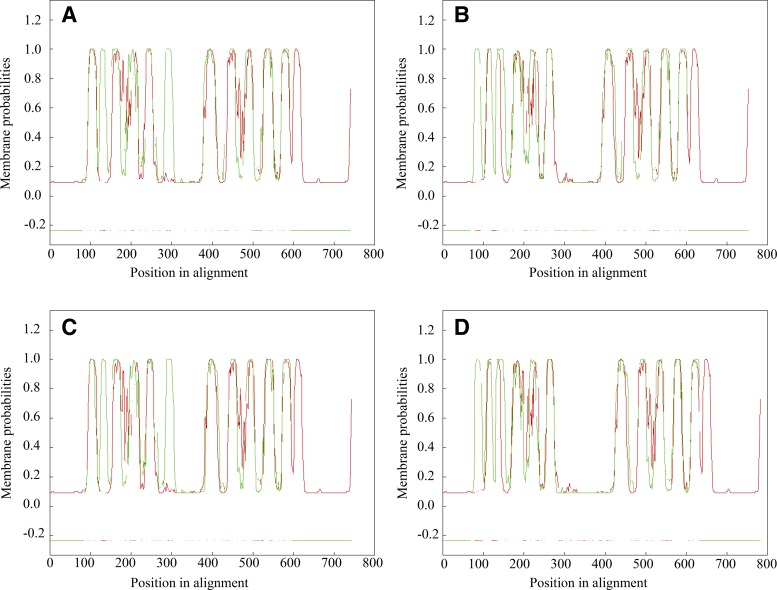
Pairwise alignment of the transmembrane domains of Si efflux transporters. Pairwise alignment of the transmembrane domains predicted in *Rn*Slc34a2 rat protein (red) with the four Si efflux transporters in plants (green). Transmembrane domains were predicted by OCTOPUS (56), and subsequent alignment was performed by AlignMe tool (51). *A*: *Os*Lsi2 (rice); *B*: *Zm*Lsi2 (maize); *C*: *Hv*Lsi2 (barley); *D*: *Cm*Lsi2–1 (pumpkin).

Comparative sequence analysis of Slc34a2 indicated no significant homology with known plant or algal Si transporters. However, marked similarities were revealed upon pairwise alignment of the transmembrane domains of Slc34a2 and the plant Si efflux transporter Lsi2 (Fig. 7), thereby suggesting a conserved structure among Si efflux proteins.

### Phylogenetic Analysis of the Slc34 Family

Sequence alignment within the rat Slc34 gene family led to the identification of a ~30-residue stretch that is only present in Slc34a2. Given that Slc34a1 and Slc34a3 were not upregulated under Si deprivation, this finding points towards the possibility that the ~30-residue stretch conveys Si transport activity to Slc34a2 (Fig. 1*C* and Table 2).

**Table 2.** Predicted transmembrane domains of Slc34a2

Helix No.	NH_2_ Terminus	Transmembrane Region	COOH Terminus	Helix Type	Length, a.a.
1	95	FQGIGKFILLLGFLYLFVCSLDV	117	1°	23
2	137	NSIMSNPVAGLVIGVLVTVMVQS	159	2°	23
3	166	IIVSMVASSLLSVRAAIPIIMGA	188	2°	23
4	374	LILCGCLIMIVKLLGSVLRGQVA	396	1°	23
5	422	VGAGMTFIVQSSSVFTSAMTPLI	444	1°	23
6	459	LGSNIGTTTTAILAALASPGNTL	481	2°	23
7	527	WFAVFYLIFFFLLTPLTVFGLSL	549	1°	23
8	555	LVGVGVPIILLILLVLCLRMLQA	577	1°	23
9	618	**CCCCCRVCCRVCCMVCGCKCCRC**	640	2°	23

The transmembrane domains were predicted using SOSUI software. The sequence highlighted in yellow through multiple sequence alignment of the three *Rattus norvegicus* Slc34 family members (Fig. 1*C*) is present in the ninth transmembrane helix of Slc34a2 (shown in bold). The COOH-terminal and NH_2_-terminal amino acids for each transmembrane domain are indicated, as is the type of α-helical structure (i.e., primary or secondary helices, denoted as 1° and 2°, respectively).

**Fig. 8.**
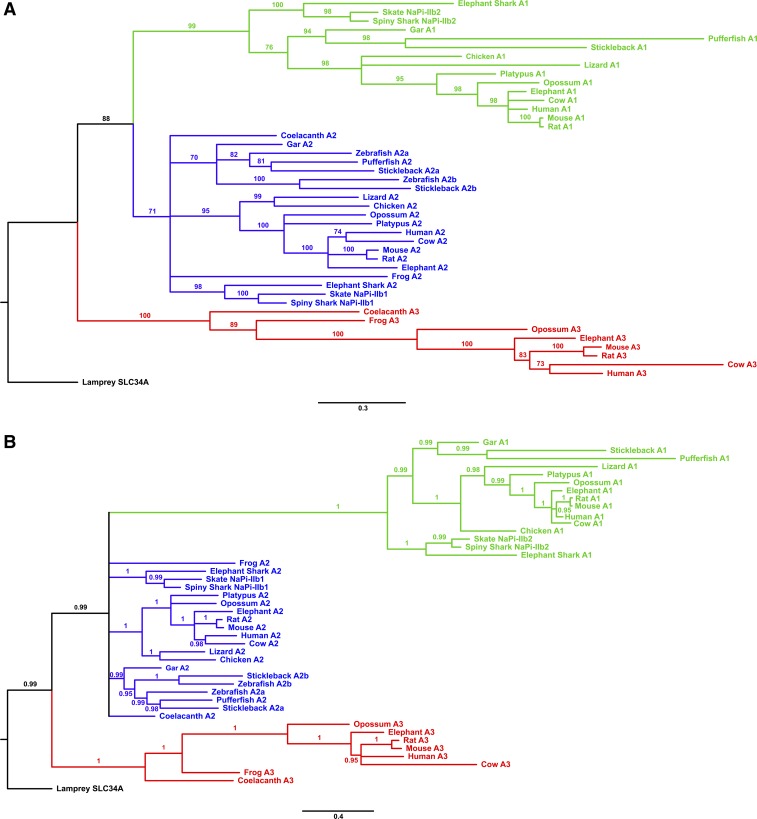
Phylogeny of Slc34a gene family member in vertebrates. *A*: the tree was produced using PhyML maximum likelihood analysis with the JTT+G+I model from an alignment of 880 positions. Numbers at nodes are a percentage of 100 bootstrap replicates, with nodes having <70% bootstrap support being collapsed. *B*: the tree was produced using Phylobayes Bayesian MCMC analysis under the CAT +G+I model (15 parallel chains with sampling every 100 cycles, burn-in one-fifth the total size of the chain) from an alignment of 880 positions. Numbers at nodes indicate posterior probabilities, with nodes having <0.95 support being collapsed. The scale bar indicates the average number of amino acid substitutions per site. The Slc34a1 clade is in green, the Slc34a2 clade is in blue, and the Slc34a3 clade is in red. The trees are rooted using the single Slc34a homolog identified from the lamprey genome. The Slc34a gene phylogeny largely agrees with the species phylogeny for vertebrates (33), with incongruent branches (e.g., the basal branches of the a2 clade) only having low statistical support. The maximum likelihood phylogenetic analyses resolve that the Slc34a clade evolved from a single ancestor in jawless vertebrates, and likely involved two main duplication events, initially producing the a3 and a1+2 clades, with a further divergence of the a1 and a2 clades. A teleost-specific duplication event resulted in the evolution of Slc34a2a and Slc34a2b, as found in stickleback and zebrafish. The Bayesian analysis had poor phylogenetic resolution at the base of the a2 clade, but still resolves the a1 and a3 groups as distinct monophyletic clades, and is not incongruous with the maximum likelihood analysis results. For full details of the species and sequences used see Supplemental Table S1.

**Fig. 9.**
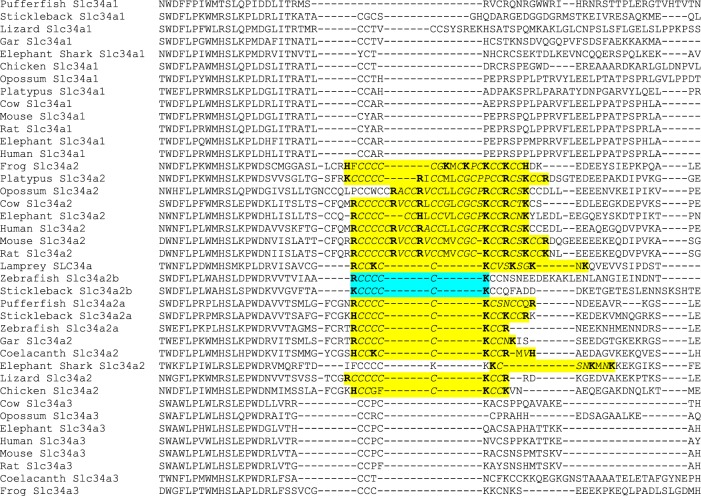
Alignment of vertebrate Slc34a protein sequences showing characteristic motif conserved across members of the Slc34a2 group. The alignment shows the region around the portion identified as unique to rat Slc34a2 in comparison to rat Slc34a1 or Slc34a3 (see Fig. 1*C*). Highlighted in yellow are the homologous regions in other vertebrate Slc34a2 proteins, and in the Slc34a-type lamprey sequence. The characteristic Slc34a2 motif identified within this region contains at least three positive residues uninterrupted by any negatively charged residues, with the positive residues regularly spaced apart by at least four small residues (primarily cysteines). A Slc34a sequence containing this motif was found in all vertebrate species investigated. The only members of the Slc34a2 clade (see Fig. 8) where this motif was incomplete was are in the zebrafish and stickleback SLC34a2b (highlighted in blue). Positively charged residues are shown in bold and small amino acids are in italics. Sequence names correspond to the species and gene identifiers given in Supplemental Table S1 and to the phylogeny shown in Fig. 8. The incomplete spiny shark and skate NaPi-IIb sequences are omitted due to this region being missing from the EMBL/GenBank data. The alignment was generated using MUSCLE (http://www.ebi.ac.uk/Tools/msa/muscle/).

Phylogenetic analysis of the Slc34a genes from a range of vertebrates (Supplemental Table S1 and Fig. 8) showed that the family underwent an expansion relatively early in vertebrate evolution, resulting in three distinct main groups (a1, a2, and a3) among the modern jawed vertebrates. At least one member of the a2-group was found in all of the jawed vertebrate genomes searched. In contrast, losses of the a1- and a3-group genes were observed in several fully sequenced genomes (e.g., zebrafish, chicken). This would suggest that the Slc34a2-group genes have a unique or important role whose loss cannot be complemented for by other transporters, and that this function is conserved across the jawed vertebrates. Common to all members of the Slc34a2 group, and to the homologous Slc34a2 gene in the lamprey, is a motif containing three positive amino acid residues (R, H, or K) separated by smaller uncharged residues (commonly C or S) (Fig. 9). This motif aligns with the unique predicted transmembrane domain noted above in the rat Slc34a2 gene (Fig. 1*C* and Table 2), and points towards an important functional role. Conserved positively charged amino acids have been noted in other Si-related proteins, such as the GRQ motifs of the SIT active Si transporters (32, 51). It may be postulated that these residues interact with local negative charges on the silicic acid molecule as part of a general biochemical basis for transmembrane Si transport.

### Conclusions

The identification that Slc34a2 can transport Si in mammals establishes another distinct gene family of Si transporters that could be involved in the regulation of Si homeostasis and that bears no sequence similarity with known Si-related genes in plants, sponges, choanoflagellates, or diatoms, although it shows strong structural similarities to silicon exporters in plants. Crucially, our work is also one of the first pieces of evidence for a functionally relevant Si-responsive gene in mammals. In parallel with this work, Garneau et al. (18) and Deshmukh et al. (8) have recently identified Si-permeable aquaporins that appear to play an important role in Si influx. Coupled with the active efflux transporter that is reported herein, we propose a Si transport model in mammals that mirrors that known in plants (28), i.e., a model in which an influx and efflux transporter must be present to allow Si movement through cells. Here, Slc34a2 is effluxing H_4_SiO_4_ from the renal tubular epithelial cell into the circulation, i.e., it is involved in the reabsorption of H_4_SiO_4_ in the kidneys. As an inevitable consequence of this expression at this cellular location, phosphate will be moved in the opposing direction. Dietary Si-P interactions have been noted, with animals on a Si-deficient diet showing conserved bone Si levels but depleted bone P levels (24). Assuming Slc34a2 is similarly involved in bone conservation of Si as it is in the kidney then our results explain these observation (24). Finally, it is also interesting to note that Lsi2’s are equally upregulated in plants in conditions of Si deprivation (34), a phenomenon that was instrumental in identifying Slc34a2 in this work. Collectively, our data provide indication that, rather than being a biochemically inert element, Si in fact plays a role in vertebrate physiology deserving of its preservation under exposure conditions of deprivation.

## GRANTS

This work was supported by Medical Research Council Grant MC_US_A090_0008/Unit Programme number U1059) (to J. J. Powell); Charitable Foundation of the Institute of Brewing and Distilling, UK (to S. Ratcliffe and R. Jugdaohsingh); Grant-in-Aid for Scientific Research on Innovative Areas from the Ministry of Education, Culture, Sports, Science and Technology of Japan (No. 22119002) (to J. F. Ma and N. Mitani-Ueno); Biotechnology and Biological Sciences Research Council (BBSRC) Comparative Genomics Training Grant BB/E527604/1 and a Leathersellers’ Company Scholarship awarded by Fitzwilliam College, Cambridge (to A. Marron); Natural Sciences and Engineering Research Council of Canada (No. 364175) and Canada Foundation for Innovation (No. 950-205342) (to J. Vivancos, R. Deshmukh, and R. R. Bélanger); Netherlands Nutrigenomics Centre (to M. V. Boekschoten and M. Müller); Natural Sciences and Engineering Research Council of Canada (to S. D. Kinrade); and Canadian Institutes of Health Research (to P. Isenring).

## DISCLOSURES

No conflicts of interest, financial or otherwise, are declared by the author(s).

## AUTHOR CONTRIBUTIONS

S.R., R.J., J.V., A.M., R.D., N.M.-U., J.R., and M.V.B. performed experiments; S.R., R.J., J.V., A.M., J.F.M., N.M.-U., J.R., J.W., and M.V.B. analyzed data; S.R., R.J., R.D., J.R., J.W., R.C.M., S.D.K., P.I., R.R.B., and J.P. interpreted results of experiments; S.R., R.J., A.M., R.D., J.R., J.W., and R.C.M. prepared figures; S.R., R.J., and J.P. drafted manuscript; S.R., R.J., J.V., A.M., R.D., J.F.M., N.M.-U., M.V.B., R.C.M., S.D.K., P.I., R.R.B., and J.P. edited and revised manuscript; S.R., R.J., J.V., A.M., R.D., J.F.M., N.M.-U., M.V.B., M.M., R.C.M., S.D.K., P.I., R.R.B., and J.P. approved final version of manuscript.

## Footnotes

1 Gene array data have been submitted to the Gene Expression Omnibus repository and assigned the reference GSE58404.
